# Dural Arteriovenous Fistula in Neuro-Behçet's Disease: Association or Chance?

**DOI:** 10.7759/cureus.54988

**Published:** 2024-02-26

**Authors:** Yuka Nakaya, Koji Hayashi, Norichika Hashimoto, Asuka Suzuki, Shiho Mitsuhashi, Mamiko Sato, Kouji Hayashi, Yasutaka Kobayashi

**Affiliations:** 1 Department of Rehabilitation Medicine, Fukui General Hospital, Fukui, JPN; 2 Department of Neurosurgery, Fukui General Hospital, Fukui, JPN; 3 Graduate School of Health Science, Fukui Health Science University, Fukui, JPN

**Keywords:** neuro-behçet's disease, behçet’s disease, arteriovenous malformations, arteriovenous (av) fistula, dural arteriovenous fistula (davf)

## Abstract

Behçet's disease (BD) is a multisystemic vasculitis disorder. Neuro-Behçet's disease (NBD) is a set of neurologic symptoms imputable to an underlying Behçet vasculitis. Among the wide range of vascular abnormalities secondary to BD, a dural arteriovenous fistula (dAVF) is not classically described. Whether a dAVF is associated with BD or dAVF is a chance occurrence is still a matter of debate. Herein, we describe an NBD case of a 48-year-old male, presenting with headache and fever, where a dAVF was seen on imaging. He was treated with prednisolone and colchicine, followed by the surgical resection for dAVF. Then, we discuss the possible association between BD and dAVF based on the latest literature.

## Introduction

Behçet's disease (BD) is a multisystemic vasculitis disorder and its etiology is almost unknown [1]. BD can affect small and large vessels in both veins and arteries [1]. Indeed, BD has risks of developing multiple vessel-related complications, including thromboses, stenoses, occlusions, and aneurysms throughout the body [2]. Classically, the symptoms of BD are characterized by recurrent oral aphthae, genital ulcers, variable skin lesions, arthritis, uveitis, and thrombophlebitis [1]. In addition, the gastrointestinal system and central nervous system (CNS) symptoms are noted in some cases [1].

According to the Consensus Status Agreement, neuro-Behçet's disease (NBD) is defined as the neurological predominant symptoms of patients who have suffered or are suffering from other symptoms related to BD [1,3]. Its neurological symptoms can affect both the CNS and peripheral nervous system (PNS), but the CNS is more affected rather than the PNS [1]. In the CNS, vascular involvement is also reported, including dural sinus thrombosis, intracranial and extracranial aneurysm formation, and arterial vasculitis [1]. In addition, a few reports have been published about dural arteriovenous fistulas (dAVFs) related to BD [4-6], but no conclusion has been reached as to whether a dAVF is related to BD or a chance occurrence. In this report, we describe a case of NBD accompanied by a dAVF and summarize our consideration on the following question: is it association or chance occurrence?

## Case presentation

A 48-year-old Japanese male with a past medical history of meningitis at 20 years old and diabetes mellitus presented to the emergency department for headaches. His sister also was known for meningitis at 20 years old. He was diagnosed with tension headache and was discharged home with a non-steroidal anti-inflammatory drug (NSAID) prescription. First, his headaches improved, but he consulted again one month later for worsening headaches. Vital signs revealed normal results of body temperature (36.5 degrees Celsius), blood pressure (107/75 mmHg), and pulse rate (88 beats/minute). Physical examination revealed no vulvar ulcer, but oral aphthosis and a folliculitis-like rash. No ophthalmic symptoms were observed. Neurological exam was unremarkable, including nuchal rigidity, neck flexion test, paralysis or sensory disturbance, except mild jolt accentuation, aspontaneity, and short-term memory impairment. Blood tests revealed elevated white blood cell, gamma-glutamyl transpeptidase, cholinesterase, glucose, hemoglobin A1c, urea nitrogen, creatinine, C-reactive protein, fibrinogen, D-dimers, erythrocyte sedimentation rate, and protein C activity (Table 1).

**Table 1:** Results of blood tests on admission.

Inspection items	Result	Reference range	Inspection items	Result	Reference range
White blood cell count	12200 /μl	(3300–8600)	Low-density lipoprotein cholesterol	164mg/dl	(70–140)
Red blood cell count	539×10⁴ /μl	(386–492×10⁴)	High-density lipoprotein cholesterol	41 mg/dl	(40–70)
Hemoglobin	15.4 g/dl	(11.6–33.4)	Triglyceride	188 mg/dl	(30–149)
Blood platelet	24.8×10⁴ /μl	(15.8–34.8)	Prothrombin time	11.3 sec	(10.0–13.0)
Total protein	7.7 g/dl	(6.6–8.1)	Prothrombin time-international normalized ratio	0.96	(0.85–1.15)
Albumin	4.4 g/dl	(4.1–5.1)	Activated partial thromboplastin time	34.0 sec	(25.0–40.0)
Total bilirubin	1.0 mg/dl	(0.4–1.5)	Fibrinogen	594.1 mg/dl	(150–400)
Alkaline phosphatase	308 U/l	(106–322)	D-dimmer	7.0 μg/ml	(<1.0)
Aspartate aminotransferase	24 U/l	(13–30)	Erythrocyte sedimentation rate	77 mm/hr	(3-15)
Alanine aminotransferase	25 U/l	(7–30)	Antinuclear Antibody	1:<40	
Lactate dehydrogenase	163 U/l	(124–222)	Rheumatoid factor	2.9 IU/mL	(<15)
Creatine kinase	45 U/l	(60–230)	anti-SS-A antibody	<0.5 U/ml	(<10)
γ-glutamyl transpeptidase	77 IU/l	(<50)	anti-SS-B antibody	<0.5 U/ml	(<7)
Cholinesterase	450 U/l	(213–501)	Myeloperoxidase-Antineutrophil Cytoplasmic Antibody	<0.5 IU/ml	(<3.5)
Glucose	194 mg/dl	(73–109)	Proteinase 3-Antineutrophil Cytoplasmic Antibody	<0.5 IU/ml	(<3.5)
Hemoglobin A1c	7.8%	(<5.5%)	Immunoglobulin G anticardiolipin antibody	1.0 U/ml	(<12.3)
Blood urea nitrogen	35.4 mg/dl	(8.0–20.0)	Protein C activity	177%	(64–166)
Creatinine	1.48 mg/dl	(0.46–0.79)	Protein S activity	127%	(67–144)
Natrium	130 mmol/l	(138–145)	Soluble interleukin-2 receptor	403 U/ml	(121–623)
Potassium	4.2 mmol/l	(3.6–4.8)	β-D-glucan	3.2 pg/ml	(<20)
Chlorine	95 mmol/l	(101–108)	Procalcitonin	0.03 ng/ml	(<0.05)
C-reactive protein	1.62 mg/dl	(0.00–0.14)	Human leukocyte antigen A26	positive	

Microbiological tests of blood revealed a previous infection pattern of herpes simplex virus, varicella-zoster virus, cytomegalovirus, and Epstein-Barr virus and that he was less likely to have been infected with influenza A and B, adenovirus, or Candida (Table 2).

**Table 2:** Results of the microbiological tests on admission.

Inspection items	Result
Anti-human immunodeficiency virus antibody	Negative
Anti-herpes simplex virus immunoglobulin G antibody	Positive
Anti-herpes simplex virus immunoglobulin M antibody	Negative
Anti-varicella-zoster virus antibody immunoglobulin G antibody	Positive
Anti-varicella-zoster virus antibody immunoglobulin M antibody	Negative
Anti-mumps immunoglobulin G antibody	Positive
Anti-mumps immunoglobulin M antibody	Negative
Anti-cytomegalovirus immunoglobulin G antibody	Positive
Anti-cytomegalovirus immunoglobulin M antibody	Negative
Epstein-Barr virus anti-viral capsid antigens immunoglobulin G antibody	Positive
Epstein-Barr virus anti-viral capsid antigens immunoglobulin M antibody	Negative
Epstein-Barr virus anti-nuclear antigen immunoglobulin G antibody	Positive
Influenza virus A antigen	Negative
Influenza virus B antigen	Negative
Adenovirus antigen	Negative
Candida antigen	Negative

Cerebrospinal fluid (CSF) tests revealed pleocytosis and elevated protein and interleukin-6 levels but were negative for CSF culture or cytology (Table 3).

**Table 3:** Results of the cerebrospinal fluid tests on admission.

Inspection items	Result	Reference range
White blood cell count	354 /μl	(<5)
Mononuclear cell	289 /μl	
Polynucleosis	65 /μl	
Protein	84.6 mg/dl	(<40)
Sugar	94 mg/dL	
Immunoglobulin G index	0.56	(<0.73)
Interleukin-6	101 pg/ml	(<17.0)
Angiotensin-converting enzyme	0.3 U/l	(8.3–21.4)
Myelin basic protein	<31.2 pg/ml	(<102.0)
Oligoclonal band	Negative	
Cerebrospinal fluid culture	Negative	
Cerebrospinal fluid cytology	Negative for tumor cell	

**Figure 1:**
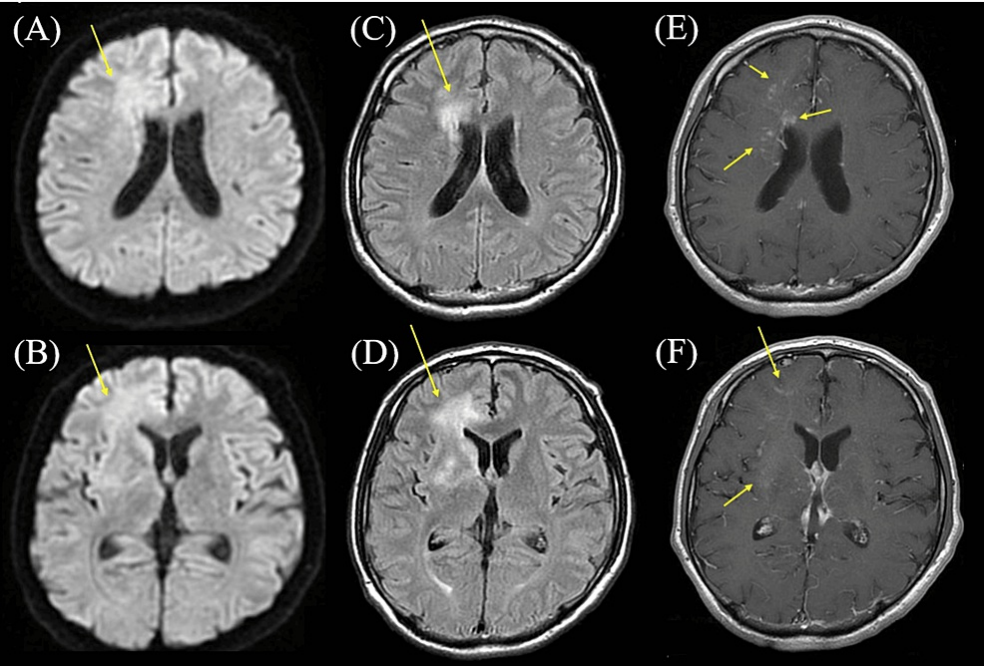
Results of the brain magnetic resonance imaging (MRI). (A, B) Diffusion-weighted brain MRI showing hyperintensity in the right frontal lobe (arrowhead). (C, D) T2-fluid attenuated inversion recovery (FLAIR) brain MRI showing hyperintensity in the right frontal lobe (arrowhead). (E, F) Gadolinium-enhanced T1-weighted brain MRI showing enhanced lesion in the area of hyperintensity by diffusion-weighted and T2-FLAIR MRI.

**Figure 2:**
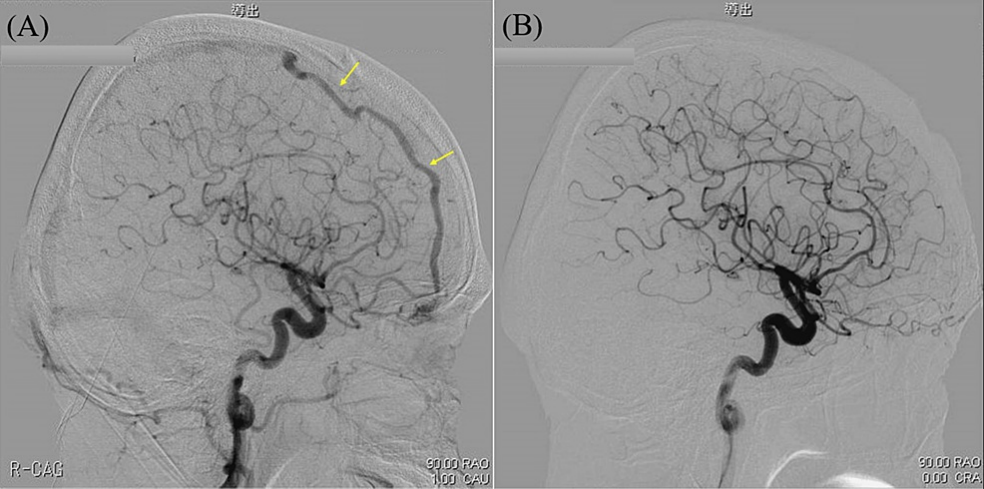
Results of the cerebral angiography (CAG). (A) CAG on admission showing an ethmoidal dural arteriovenous fistula (dAVF) (arrowhead). (B) CAG on six months after operation showing decreased density and thinning of dAVF on radiography but persisted dAVF (arrowhead). (C) CAG on one and a half years after starting immunotherapy and surgical intervention showing the disappearance of the dAVF.

Gadolinium-enhanced T1-weighted brain magnetic resonance imaging (MRI) showed a white matter lesion in the right frontal lobe (Figure 1A-1D), and its lesion had a contrast-enhanced effect (Figure 1E, 1F). Cerebral angiography (CAG) revealed a dAVF (Figure 2A).

We classified the dAVF in our case into Cognard type IIb. Genetic testing disclosed HLA-A*26 positivity. Based on the revised diagnostic criteria proposed by the BD Research Committee of Japan, we diagnosed him with incomplete BD, and the patient was diagnosed with NBD by the International Consensus Recommendation (ICR) criteria for NBD diagnosis. He was treated with prednisolone (starting dose: 30 mg/day) and colchicine (starting dose: 1.5 mg/day). In addition, surgical resection was performed for the dAVF. One and a half years after the operation, the dAVF disappeared (Figure 2B), and other symptoms were well-controlled by immunotherapy. No recurrence had been observed for four years after the operation.

## Discussion

This report describes a rare case of NBD accompanied by a dAVF. He was diagnosed with incomplete BD and NBD by diagnostic criteria. Regarding the dAVF, the feeding artery was from the ethmoidal artery. After surgical resection and immunosuppressant therapy, the dAVF disappeared and his symptoms were well-controlled.

BD is a multi-systemic vasculitis disorder that affects small and large vessels and involves both the veins and arteries [1]. Some diagnostic criteria have been proposed; for instance, the revised Japanese diagnostic criteria for BD were published in 2004 [7]. According to these criteria, patients with four main symptoms (recurrent aphthous ulcers on the oral mucosa, skin lesions, ocular lesions, and genital ulcer) were defined as complete BD; patients with three main symptoms, two main symptoms, and two additional symptoms, typical ocular lesions with one main symptom, or typical ocular lesions with two additional symptoms were defined as incomplete BD. Our case meets two main symptoms including recurrent aphthous ulcers on oral mucosa and skin lesions and two additional symptoms including vascular lesions and CNS lesions, so we diagnosed him with incomplete BD. In addition, HLA-A*26 was positive in our case. It is reported that HLA-A*26 is one of the causative haplotypes of BD [8]. On the other hand, for the diagnosis of NBD, the ICR criteria are widely used [3]. This diagnostic tool consists of the following three criteria: 1) satisfy widely accepted BD criteria, 2) neurological syndrome caused by BD and characteristic abnormalities supported by neuroimaging and/or CSF, and 3) no better explanation for the neurological findings. In our case, the patient developed short-term memory impairment, the brain lesion in the right frontal lobe was noted with contract-enhanced effect, and CSF findings were typical for NBD, including elevated cell count, protein, and interleukin-6. Based on these findings, we diagnosed him with definite NBD.

BD patients can develop multiple vessel-related complications, including thromboses, stenoses, occlusions, and aneurysms [2]. In addition, a few reports mentioned that BD can be related to arteriovenous fistula (AVF) [4-6,9,10]. As far as we know, it has been reported that AVFs occur in three locations of the body part in patients with BD: the renal AVF, the hepatic artery to the superior mesenteric vein, and dAVFs. Alamlih et al. reported a renal arteriovenous fistula (RAVF) in a patient with BD [9]. Regarding the possible cause of the genesis of RAVF, the authors mentioned the rupture of the renal aneurysm into the renal vein [9]. In addition, Cekirge et al. reported AVF in the hepatic artery, the superior mesenteric vein, and its etiology was reported as the rupture of an aneurysm [10]. On the other hand, a dAVF associated with NBD was first reported in 1980 by Imaizumi et al. [6]. The authors described the combination of NBD with a dAVF would seem to be a chance occurrence [6]. Subsequently, a few case reports have been published about both NBD and dAVFs [4-6]. Notably, Falgàs et al. reported a recurrent dAVF in a patient with BD [4]. The patient in the previous report had two dAVFs in the posterior fossa and superior sagittal sinus on the first admission, and one year after the treatment of two dAVFs, another two dAVFs were developed [4]. Although the possible etiology of developing dAVFs was not mentioned in the previous report, the recurrence of a dAVF after treatment suggests that certain mechanisms by BD can induce a dAVF. Therefore, dAVFs were considered to be associated with NBD rather than a chance occurrence.

We speculated three pathways in developing dAVFs in patients with NBD. First is an aneurysm pathway. Like RAVF or artery-to-portal vein fistula [9,10], the aneurysm rupture into a near vein might develop dAVFs. However, no aneurysm was observed here. Second is a thrombophlebitis pathway. In BD, it is reported that fistulas can be developed in the presence of inflammation and infection [11]. Thrombophlebitis by NBD can increase venous pressure and cause inflammation and might affect venous flow and artery, leading to forming dAVFs. Third is a sinus thrombosis pathway. NBD can cause sinus thrombosis [12]. Moreover, sinus thrombosis can induce chronic venous hypertension, leading to developing dAVFs at near and/or remote vessels from the sinus [13]. Although we speculated the above three pathways, the lack of pathophysiological evidence is a limitation of this paper. Whereas the mechanism of developing dAVFs remains unclear, further studies are needed to reveal the underlying mechanisms of the development of dAVFs in NBD.

## Conclusions

We presented a rare case of NBD with a dAVF. We suggest that a dAVF is not a chance occurrence but is related to NBD. Further studies are needed to reveal the underlying mechanisms of the development of dAVFs in NBD.
